# Left Accessory Mental Foramen in Dry Mandibles in Department of Anatomy of a Medical College: A Descriptive Cross-sectional Study

**DOI:** 10.31729/jnma.7770

**Published:** 2022-09-30

**Authors:** Nripendra Tiwari

**Affiliations:** 1 Department of Anatomy, Kathmandu Medical College and Teaching Hospital, Duwakot, Bhaktapur, Nepal

**Keywords:** *mandible*, *mental foramen*, *mental nerve*

## Abstract

**Introduction:**

The presence of accessory mental foramen is one of the anatomical variations in the mandible. The occurrence of accessory mental foramen can differ in terms of number, shape, and size or there can even be an absence of it. The aim of this study was to find out the prevalence of left accessory mental foramen in dry mandibles in Deparment of Anatomy of a medical college.

**Methods:**

This descriptive cross-sectional study was conducted in a medical college among dry mandibles of a medical college from 10 January 2021 to 10 June 2021. Ethical approval was obtained from the Institutional Review Committee (Reference number: 207202005). Intact dry mandibles were studied for the prevalence of left accessory mental foramen. A convenience sampling technique was used. Point estimate and 90% Confidence Interval were calculated.

**Results:**

Among 47 dry mandibles, the prevalence of left accessory mental foramen was found to be four (8.51%) (1.81-15.21, 90% Confidence Interval). The mean diameter of the left accessory foramen was found to be 1.02±0.03 mm.

**Conclusions:**

The prevalence of left accessory mental foramen in mandibles was similar to the studies done in similar settings.

## INTRODUCTION

Accessory mental foramen is the anatomical variation of the mandible. The bilateral presence of mental foramen is an oval or circular opening located on the anterolateral aspect of the mandible. It provides a path to mental nerves and vessels.^1^ The paralysis of the mental nerve is one of the principal complications of surgeries on the mental foramen region.^2^ Accessory mental foramen is found due to branching of the mental nerve before its passage through the mental foramen.

The presence of accessory mental foramen can cause surgical difficulties and neurosensory complications during open reduction of mandibular fractures, segmental orthognathic surgeries and implant surgeries.^3^ Preoperatively verification of the presence of accessory mental foramina would prevent injury to accessory nerve and complications.^4^ Particular attention preoperatively should be given to the probable occurrence of the accessory mental foramen to avoid neurovascular complications.^5,6^

The aim of this study was to find the prevalence of left accessory mental foramen in the dry mandible in a medical college.

## METHODS

This descriptive cross-sectional study was conducted in the Department of Anatomy from 10 January 2021 to 10 July 2021 after obtaining ethical clearance from the Institutional Review Committee of Kathmandu Medical College and Teaching Hospital, Duwakot, Bhaktapur, Nepal (Reference number: 207202005). The study included all the dry mandibles irrespective of age and sex. The wet, decayed, damaged mandibles with loss of important anatomical structures like condylar, coronoid and alveolar processes were excluded from the study.

Convenience sampling technique was used and sample size calculated according to the following formula:


$$\begin{array}{ll} \text{n} & ={\text{Z}}^{2}\times \frac{\text{p}\times \text{q}}{{\text{e}}^{\text{2}}}\\ & =1.96^{2}\times \frac{0.6\times 0.4}{0.1^{2}}\\ & =65 \end{array}$$

Where,

n = minimum required sample size
Z = 1.645 at 90% Confidence Interval (CI)
p = prevalence of left accessory mental foramen, 60%^6^
q = 1-p
e = margin of error, 10%

The calculated sample size was 65. As the study population was finite was 47. Now correcting the sample size for finite population:


$$\begin{array}{ll} \text{n'} & =\frac{\text{n}}{1+\frac{\text{n}-1}{\text{N}}}\\ & =\frac{\text{65}}{1+\frac{\text{65}-1}{\text{47}}}\\ & =28 \end{array}$$

Where,

n'= adjusted sample size
N= finite population
n= calculated sample size

The adjusted sample size was 28. However, a sample size of 47 was taken for the study. The dimension was measured using 0.01 mm sensitive digital callipers. Data was collected by a self-designed questionnaire in a written form to obtain the necessary information on the variables of the study. The number of mandibles obtained were labelled and observed in detail and photographed for easy description.

The collected data was compiled in Microsoft Excel 2013 and further analysed in IBM SPSS Statistics 20.0. Point estimate and 90% CI were calculated.

## RESULTS

Among 47 dry mandibles, the prevalence of left accessory mental foramen was found to be four (8.51%) (1.81-15.21, 90% CI). The prevalence of oval shaped left accessory mental foramen was found to be 3 (75%) (Table 1).

**Table 1:** Shape of left accessory mental foramen (n= 4).

Shape	n (%)
Oval shape	3 (75)
Round shape	1 (25)

The mean diameter of the left accessory mental foramen was found to be 1.02±0.03 mm (Table 2).

**Table 2:** Location of left accessory mental foramen with its diameter and mean in the study population of dry adult human mandible (n= 4).

Parameters	Mean±SD
Diameter of left accessory mental foramen	1.02±0.03 mm
Antero-posterior distance between left accessory mental foramen and left mental foramen	5.01±0.40 mm
Vertical distance between left accessory mental foramen and left mental foramen	2.76±0.19 mm
	

## DISCUSSION

The results of the present research showed that the left accessory mental foramen was prevalent in 4 (8.51%). Similar result was found with prevalence of 11.5% in a study using cone beam computed tomography.^7^ As per the research carried out on Indian adult human mandible,^8^ the prevalence of left accessory mental foramen was found to be 8%. The result is in contrast with those of the studies carried out on Israeli population,^9^ where the prevalence was found to be only 2.8%. The cause for the difference in result could be due to differences in the sample size, age, sex, dentulous condition of the study population and use of cone beam computed tomography. The mean diameter of left accessory mental foramen was found to be 1.07 mm. Similar results were found by the research carried out in Kathmandu valley population, Nepal^10^ with average diameter of 1mm for mean diameter of left accessory mental foramen. The oval shape of left accessory mental foramen was found to be in 3 (75%) and round shape of it in 1 (25%) among 47 dry mandibles. The result is in contrast with the study carried in dry human mandibles of South Indian population^11^, where the round shape of accessory mental foramen was found to be in majority of 74%. The mean vertical distance from alveolar crest margin to centre of left mental foramen in the present study was found to be 11.57±1.08 mm. Similarly, the mean vertical distance from base of the mandible to the centre of left mental foramen was found to be 13.41±1.25 mm.

The mean vertical distance between left accessory mental foramen and left mental foramen was found to be 2.76±0.19 mm. These results are however similar with the study conducted in mandibles of the same South Indian population^11^ with the situation of accessory mental foramen at a mean distance of 2.96 mm from mental foramen and 11.24 mm from lower border of the body of the mandible. Similar results with a slight difference were also found in research conducted in another research carried over South Indian population.^12^

The cause for the difference in the result could be due to difference in the total sample size of 90 dry adult mandibles, irrespective of the age and sex with either all the teeth intact or with preserved alveolar margins and difference in visual examination of number, shape and evaluation of mental and accessory mental foramen from individual to individual.

All the measurements by using various parameters and instrumentations were recorded by the principal author only and dry adult human mandibles were labelled and photographed to reduce the biases during the research work. The use of cone beam computed tomography (CBCT) was not considered because the unavailability of it in our set up would be the limitation of the current study.

## CONCLUSIONS

The prevalence of left accessory mental foramen in mandibles was similar to the research done in similar settings. The prior verification on existence of accessory mental foramen would prevent an accessory mental nerve injury, insufficient local anaesthesia and other surgical complications while performing surgeries.
